# Concrete Crack Detection and Classification Methods Based on Machine Vision and Deep Learning

**DOI:** 10.3390/s26082381

**Published:** 2026-04-13

**Authors:** Weibin Chen, Zhijie Peng, Xiangsheng Chen, Linshuang Zhao, Tao Xu, Qiang Li, Xianwen Huang, K. K. Pabodha M. Kannangara

**Affiliations:** 1 College of Civil and Transportation Engineering, Shenzhen University, Shenzhen 518060, China; wbchen@szu.edu.cn (W.C.); xschen@szu.edu.cn (X.C.); taoxu@szu.edu.cn (T.X.); parkli@szu.edu.cn (Q.L.); 2 Underground Polis of Academy, Shenzhen University, Shenzhen 518060, China; 3 Key Laboratory of Coastal Urban Resilience Infrastructure, Ministry of Education, Shenzhen University, Shenzhen 518060, China; 4 National Engineering Research Center of Deep Shaft Construction, Shenzhen 518060, China; 5 College of Engineering, Shantou University, Shantou 515063, China; 24zjpeng@stu.edu.cn; 6 School of Civil Engineering, Suzhou University of Science and Technology, 1701 Riverside Road, Suzhou 215009, China; huangxianwen194@usts.edu.cn; 7 Construction and Quality Management, Hong Kong Metropolitan University, Hong Kong SAR, China; kkpmkann@hkmu.edu.hk

**Keywords:** machine vision, crack detection, OTSU algorithm, Support Vector Machine, classification

## Abstract

With the rapid development of underground space, structural crack monitoring has become increasingly critical. This study proposes a unified framework integrating image preprocessing, feature extraction, model training, and safety assessment for crack analysis. An improved OTSU threshold segmentation algorithm based on sliding windows and local statistical analysis is developed to enhance noise suppression and detail preservation under complex backgrounds and varying resolutions. For crack identification and orientation classification, SVM, CNN, ResNet-18, and K-means clustering are systematically compared. The results show that the improved OTSU method outperforms the classical approach in both high- and low-resolution images. In classification tasks, SVM achieves the best performance under limited data conditions, with accuracy exceeding 96% and reaching 97% after outlier removal, outperforming CNN, K-means, and ResNet-18. Although ResNet-18 demonstrates strong overall performance with high prediction confidence across crack categories, it remains slightly inferior to SVM when training data are limited. Experimental validation using full-scale loading tests of metro shield tunnel segments further confirms the robustness of the proposed approach, with SVM achieving an accuracy of 95.45% in real-world conditions. This study provides an efficient and reliable solution for automated crack detection and classification in metro tunnel infrastructure and similar underground segment-based systems.

## 1. Introduction

As underground space development continues, the potential impact of subsurface constructions on adjacent buildings has become more pronounced, particularly regarding issues such as foundation settlement and structural displacement [1,2,3]. These problems may not only hinder normal building operations but also pose risks to urban safety [4,5,6,7]. Monitoring of cracks in underground structures holds substantial practical significance and engineering value. Real-time monitoring and analysis of cracks enable the prediction of structural failure modes and facilitate the assessment of impacts on neighboring structures, thereby supporting the scientific planning and safe management of urban underground spaces [8].

Over the past few decades, manual visual inspection, a traditional way to identify and assess cracks, has shown clear flaws in practice: it is inefficient, takes too much time, and results are subjective [9]. Although conventional manual inspection is widely used for concrete crack detection, it remains labor-intensive, time-consuming, and subjective due to heavy reliance on the experience of inspectors. It is also unsuitable for large-scale continuous monitoring and cannot precisely quantify cracks, which may affect the efficiency and reliability of structural assessment. Differences in inspector skills and experience also make results inconsistent and unstable. In monitoring the health of concrete structures, accurately identifying and assessing cracks is key to ensuring safety and durability [10]. But manual inspection, though widely used, has become increasingly problematic for these reasons. To fix these issues, digital image processing has become a better option. It automatically gets key info like whether a crack exists, where it is, and how wide it is by analyzing images of concrete surfaces. Today, main image processing methods fall into three types: Intensity threshold methods, like the OTSU algorithm, use image binarization—turning grayscale images into black and white—to separate cracks (usually darker) from the background (usually brighter) [11,12,13], yet in uneven light or with complex backgrounds (like stains or shadows), they often mistake dark spots for cracks. Edge detection methods find cracks by their edges but are sensitive to noise, creating fake edges in complex backgrounds [14,15]. Mathematical morphology, used as a helper tool, changes crack shapes with operations like dilation and erosion to improve connections or remove noise [13,16]. But choosing the right parameters depends on the situation, or else the original crack shape might get damaged.

**Figure 1:**
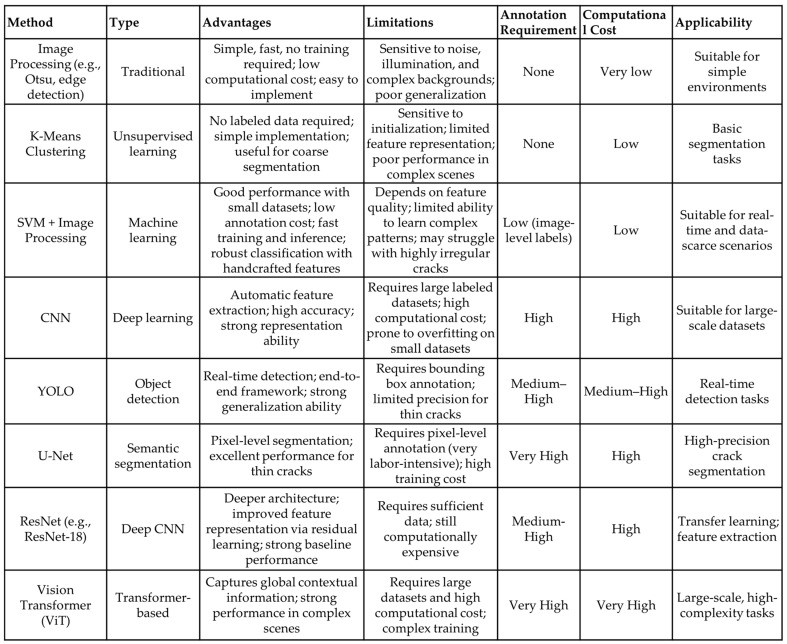
Comparison of Crack Detection and Classification Methods.

Although the aforementioned image processing methods have demonstrated great potential in crack identification, their underlying shared assumption—that the given images contain actual cracks—severely limits the realization of fully automated monitoring. In practical engineering, concrete surface images captured by digital cameras or unmanned aerial vehicles often contain both cracked and non-cracked regions (such as dark stains, shadows, dust, and lumps), and these “crack-like” features are visually indistinguishable from real cracks [17,18]. For instance, image binarization may misclassify dark stains as cracks, directly leading to an increase in the false detection rate. Therefore, accurately distinguishing real cracks from crack-like features in complex backgrounds has become a critical bottleneck in achieving fully automated crack monitoring. In recent years, the introduction of machine learning techniques has provided new insights for solving this problem; in particular, the integration of supervised learning and computer vision has significantly improved the intelligence level of crack identification. Examples include: Nyathi et al.’s [19] proposed method combining deep learning and laser calibration, which exhibited excellent performance in crack classification, segmentation, and parameter measurement accuracy, but the generalization ability of the model is limited by the scale of the training dataset; Oh et al. [20] adopted a BP neural network to realize crack extraction and detection through nonlinear classification algorithms, providing a reference for nonlinear feature modeling; Long et al. [21] proposed an algorithm combining grayscale correction and adaptive minimum error threshold segmentation, which can effectively extract cracks but lacks classification and evaluation capabilities; Nishikawa et al. [22] combined image processing with genetic programming, verifying its effectiveness under complex surface textures, though there remains room for improvement in accuracy; Han et al. [23] improved the calculation accuracy of geometric parameters by identifying crack edges and correcting parameters using an interactive genetic algorithm, yet the method involves large computational costs and performs poorly when the grayscale difference between cracks and the background is small; Li et al. [24] proposed a CNN-based classifier that achieves high-accuracy detection on large databases without manual feature extraction, highlighting the advantages of deep learning in automatic feature learning. Deep learning-based methods such as YOLO and U-Net have been widely applied to crack detection tasks. A lightweight YOLOv8-seg framework was proposed to achieve crack segmentation and quantification, improving computational efficiency while maintaining high detection accuracy [25]. Zhang et al. [26] utilized a U-Net-based fully convolutional network to perform pixel-level crack segmentation, demonstrating strong detection accuracy. Shamsabadi et al. [27] employed a Vision Transformer (ViT)-based framework for crack detection, achieving improved segmentation performance, especially for small and multi-scale cracks. However, such methods rely on dense pixel-level annotations and are therefore less suitable for data-constrained engineering scenarios. He et al. [28] proposed the ResNet architecture, which introduces residual connections to facilitate the training of deep neural networks and improve feature representation. This approach effectively alleviates the vanishing gradient problem and has become a widely used backbone in computer vision tasks. A comparison of the aforementioned crack detection methods is summarized in Figure 1.

In summary, while the evolution from traditional image processing to machine learning has gradually enhanced the automation and accuracy of crack identification, multiple technical bottlenecks persist. For traditional OTSU methods, in uneven light or with complex backgrounds (like stains or shadows), they often mistake dark spots for cracks. At a broader level, future research must address core challenges: balancing algorithm robustness with adaptability to complex scenarios, reducing reliance on large annotated datasets, and achieving full-process automation from existence detection to accurate classification.

In this study, a novel integrated framework encompassing image preprocessing, feature extraction, model training, and safety assessment was established for the comprehensive analysis of cracks in tunnel structures. To address the issues of noise interference and detail loss inherent in the traditional OTSU threshold segmentation algorithm under conditions of complex backgrounds and variable resolutions, an improved OTSU algorithm incorporating a sliding window mechanism and local statistical analysis is proposed. Subsequently, three methods—Support Vector Machine (SVM), Convolutional Neural Network (CNN), K-Means clustering and ResNet—were adopted for crack identification and orientation classification (i.e., Horizontal, Vertical, and Inclined), with a systematic comparison of their performance metrics conducted. Finally, case verification was carried out on shield tunnel segment loading tests to validate the practical applicability of the developed framework.

The main research gaps can be summarized as follows:

- Global thresholding methods lack robustness under uneven illumination and complex underground backgrounds;
- Crack-like features such as stains and shadows are difficult to distinguish from real cracks;
- Data-driven approaches rely on large annotated datasets, limiting practical applicability;
- Existing studies lack integrated frameworks for crack detection and classification.

The main contributions of this study are as follows:

- An improved OTSU algorithm incorporating sliding window and local statistical analysis is proposed;
- A comparative study of SVM, CNN, K-Means and Resnet for crack identification and orientation classification is conducted;
- A feature-based approach is proposed for accurate crack orientation recognition;
- The proposed method is validated through full-scale metro shield tunnel segment loading tests.

## 2. Methodology

In crack detection tasks, accuracy and robustness are strongly influenced by image preprocessing, feature extraction, and the choice of classification methods. In this study, the OTSU algorithm is employed for image threshold segmentation, and an improved version is further developed to enhance crack region extraction. SVM, CNN, ResNet-18, and K-means clustering are then utilized as classification models to systematically compare their performance in terms of recognition accuracy and generalization capability.

### 2.1. OTSU Threshold Segmentation Algorithm

The OTSU algorithm was proposed in 1979 by Otsu [29]. This method determines the optimal threshold by maximizing the between-class variance and has been widely applied in the field of image processing for binarization tasks. It is particularly suitable for images where there is a clear difference in grayscale distribution between the target and the background. However, its widespread use is limited in complex crack images. Relying on global gray level statistics, it falters with highly heterogeneous backgrounds—such as uneven illumination, varying textures, or noise. Unable to adapt to local gray variations, it often over-segments (misidentifying non-cracks) or under-segments (missing faint/narrow cracks) and lacks dynamic threshold adjustment for local crack features, reducing accuracy in complex surroundings [30].

### 2.2. Improved OTSU Threshold Segmentation Algorithm

To address these limitations, an improved locally adaptive thresholding method is proposed by integrating a sliding window mechanism with local statistical analysis. The method combines global thresholding with local adaptive adjustment to enhance crack detection under complex conditions. The main procedure is as follows:

(1) The classical OTSU algorithm is applied to compute the global optimal threshold $T_{1}$, which maximizes the between-class variance $\sigma_{B}^{2}(T)$ to separate the foreground and background:

(1) $$T_{1}=argmax\;\sigma_{B}^{2}(T)$$

(2) Local statistical computation: The image is divided into windows of size N×N, and the local mean $\overset{-}{I}$ and variance D within each window are calculated as:

(2) $$\overset{-}{I}\;=\;\frac{1}{N^{2}}\sum_{\left(x,y\right)\in W}I\left(x,y\right)$$

(3) $$D=\frac{1}{N^{2}}\sum_{\left(x,y\right)\in W}{\left(I\left(x,y\right)-\overset{-}{I}\right)}^{2}$$

where $I\left(x,y\right)$ denotes the grayscale value of pixel $\left(x,y\right)$ in window W, and $N^{2}$ is the total number of pixels in the window. The variance D reflects local intensity variations.

(3) Adaptive segmentation strategy: The local variance D is used to evaluate the complexity of each region. If D <τ, where τ is a predefined variance threshold, the region is considered homogeneous and segmented using the global threshold:

(4) $$T\;=\;T_{1}\;$$

Otherwise, the region is regarded as containing cracks or complex background, and a locally adaptive threshold is applied.

(4) Threshold adjustment: The local threshold is defined as:

(5) $$T_{2}=\alpha T_{1}+(1-\alpha )\overset{-}{I}$$

where $I\left(x,y\right)$ denotes the grayscale value of pixel $\left(x,y\right)$ in window W, and $N^{2}$ is the total number of pixels in the window.

Here, α acts as a weighting coefficient that balances global information and local features, allowing the threshold to adaptively adjust for improved detection of low-contrast cracks. The global threshold $T_{1}$ describes the overall intensity distribution, whereas $\overset{-}{I}$ characterizes local brightness. A larger α improves stability under uniform illumination, while a smaller α enhances sensitivity to local variations under uneven lighting and low-contrast conditions.

### 2.3. Crack Identification and Classification Using SVM

SVM is a classification algorithm based on statistical learning theory, proposed by Vapnik [31]. In this study, SVM is applied to distinguish between “crack” and “non-crack” images by constructing an optimal hyperplane, thereby enabling accurate crack identification. Due to its strong generalization ability in high-dimensional spaces, SVM is capable of effectively handling nonlinearly distributed data, making it well-suited for complex image analysis tasks such as crack detection.

In linearly separable cases, SVM constructs an optimal hyperplane to perform crack classification. The primary objective is to maximize the margin between two classes, expressed as 2/|w|. The corresponding optimization framework can be described as the hard margin model:

- Objective function:

(6) $$\;\underset{w,b}{\min}\frac{1}{2}{\left\|w\right\|}^{2}$$

- Constraint condition:

(7) $$y_{i}\left(w^{T}x_{i}+b\right)\;\geq \;1,\;\forall i$$

where w denotes the weight vector, b is the bias term, and $\left(x_{i},y_{i}\right)$ represents the training samples with $y_{i}\in \{+1,-1\}$, indicating crack and non-crack categories. The model requires that all samples strictly satisfy the classification constraint, and the optimal solution is determined by the support vectors that meet the condition $y_{i}\left(w^{T}x_{i}\;+\;b\right)\;=\;1$.

To handle noisy or non-linearly separable data, a soft margin model is introduced by incorporating slack variables $\xi_{i}\;\geq \;0$ and a regularization parameter C:

- Objective function:

(8) $$\underset{w,b}{\min}\frac{1}{2}{\left\|w\right\|}^{2}+C\sum_{i=1}^{N}\xi_{i}$$

- Constraint condition:

(9) $$y_{i}\left(w^{T}x_{i}+b\right)\;\geq \;1-\xi_{i},\;\xi_{i}\;\geq \;0,\;\forall i$$

The parameter C balances the trade-off between maximizing the margin and tolerating misclassification. A larger C imposes a heavier penalty on misclassification, while a smaller C promotes better generalization.

When crack features are not linearly separable in the original space, a kernel function mapping mechanism is employed to project the data into a higher-dimensional space. Common kernel functions include the linear kernel, Gaussian (RBF) kernel, and polynomial kernel, with respective formulations:

- Linear kernel:

(10) $$K\left(x_{i},x_{j}\right)=x_{i}^{T}x_{j}$$

- Gaussian kernel:

(11) $$K\left(x_{i},x_{j}\right)=\exp\left(-\frac{|x_{i}-x_{j}{|}^{2}}{2\sigma^{2}}\right)$$

- Polynomial kernel:

(12) $$K\left(x_{i},x_{j}\right)={\left(x_{i}^{T}x_{j}+c\right)}^{d}$$

For the linearly separable scenario, the classification function is defined as:

(13) $$f\left(x\right)\;=\;\mathrm{sign}\left(w^{T}x\;+\;b\right)$$

For kernel-based scenarios, it is formulated as:

(14) $$f\left(x\right)\;=\;\mathrm{sign}\left(\sum_{i=1}^{N}\alpha_{i}y_{i}K\left(x_{i},x\right)\;+\;b\right)$$

where $\alpha_{i}$ denotes the Lagrange multiplier, and $\mathrm{sign}\left(\cdot \right)$ indicates the predicted class label (+1 for crack, −1 for non-crack). The kernel mapping mechanism enhances the nonlinear classification capability and effectively addresses the challenge of classifying cracks when there is significant overlap with background features.

### 2.4. Crack Identification and Classification Using CNN

CNN consists of convolutional layers, pooling layers, fully connected layers, and activation functions. The convolutional layers extract local features using convolution kernels, while pooling layers down sample the feature maps to reduce dimensionality and computational complexity. Fully connected layers integrate the extracted features to perform final classification, and activation functions introduce nonlinearity, enhancing the network’s representational capacity [32].

The key components of CNNs can be described mathematically. In the convolutional layer, the input image is convolved with a kernel, expressed as:

(15) $$y(i,j)\;=\;(x\cdot w)(i,j)\;=\;\sum_{M}\sum_{N}x(i\;+\;m,\;j\;+\;n)\cdot w(m,n)$$

where x(i,j) denotes the input image or feature map, w(m,n) represents the convolution kernel of size m × n, and y(i,j) is the output of the convolution operation. The max pooling operation is defined as:

(16) y(i,j) = max{x(p,q):p∈[i,i + k], q∈[j,j + k]}

In fully connected layers, each neuron is connected to all neurons in the previous layer. The mathematical representation is:

(17) y = f(Wx + b)

where W is the weight matrix, x is the output from the preceding layer, b is the bias term, and f denotes the activation function. Activation functions such as ReLU (Rectified Linear Unit), Sigmoid, and Softmax are commonly used to introduce nonlinearity and compute class probabilities. Their formulations are as follows:

(18) ReLU(x) = max(0,x)

(19) $$\sigma (x)=\frac{1}{1+e^{-x}}$$

(20) $$\mathrm{Softmax}(x_{i})=\frac{e^{x_{i}}}{\sum_{j}e^{x_{j}}}$$

In classification tasks, the cross-entropy loss function is typically applied, defined by:

(21) $$L\;=\;-\sum_{i=1}^{C}y_{i}\log\left({\hat{y}}_{i}\right)$$

where C denotes the number of classes, $y_{i}$ is the one-hot encoded ground truth label, and ${\hat{y}}_{i}$ represents the predicted probability.

Through its multilayer convolutional structure, a CNN is capable of extracting features progressively, ranging from edge textures to high-level semantic information, thereby achieving superior performance in tasks such as image classification and object detection.

### 2.5. Fundamental Principles of Morphological Operations

Morphological operations are grounded in the theory of mathematical morphology and are designed to extract and refine shape-related features within an image [33]. In crack detection, they effectively suppress noise, enhance crack connectivity, and improve the structural integrity of segmented regions, playing an important role in both preprocessing and feature extraction stages. For crack images segmented by the OTSU method, this study applies a series of morphological operations, including dilation, erosion, opening, closing, as well as bridging and filling, to improve detection accuracy. Dilation expands crack regions to enhance connectivity and repair fragmented structures, while erosion removes isolated noise and refines edges. Opening (erosion followed by dilation) is used to eliminate small-scale noise and smooth contours, whereas closing (dilation followed by erosion) bridges discontinuities and strengthens connectivity. These operations collectively improve the quality of crack regions and provide a reliable basis for subsequent analysis [34].

In addition, morphological filling is employed to restore enclosed holes within crack regions. By improving pixel connectivity and smoothing boundaries, this operation compensates for discontinuities caused by noise, shadows, or resolution limitations, thereby enhancing the accuracy of crack skeleton extraction and ensuring stability in subsequent classification tasks.

## 3. A Novel Framework for Crack Recognition and Classification

**Figure 2:**
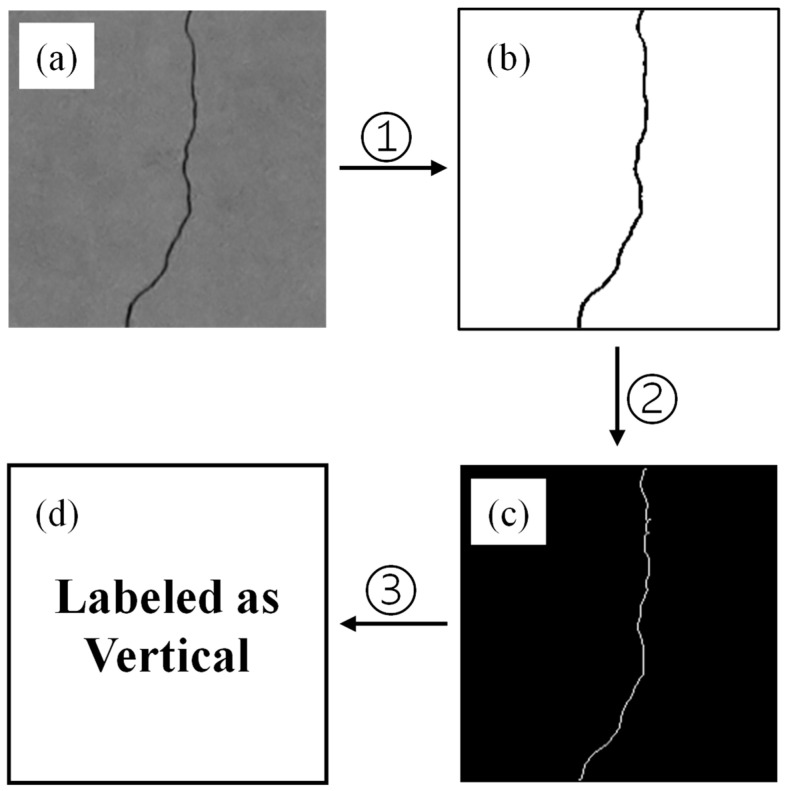
Key Image Processing and Training Set Construction: (**a**) original image, (**b**) binary segmentation image, (**c**) single-pixel image, and (**d**) Training Set; (1) improved OTSU processing, (2) morphological processing and skeletonization and (3) construct SVM training set using X, Y projection pixel histogram distribution.

**Figure 3:**
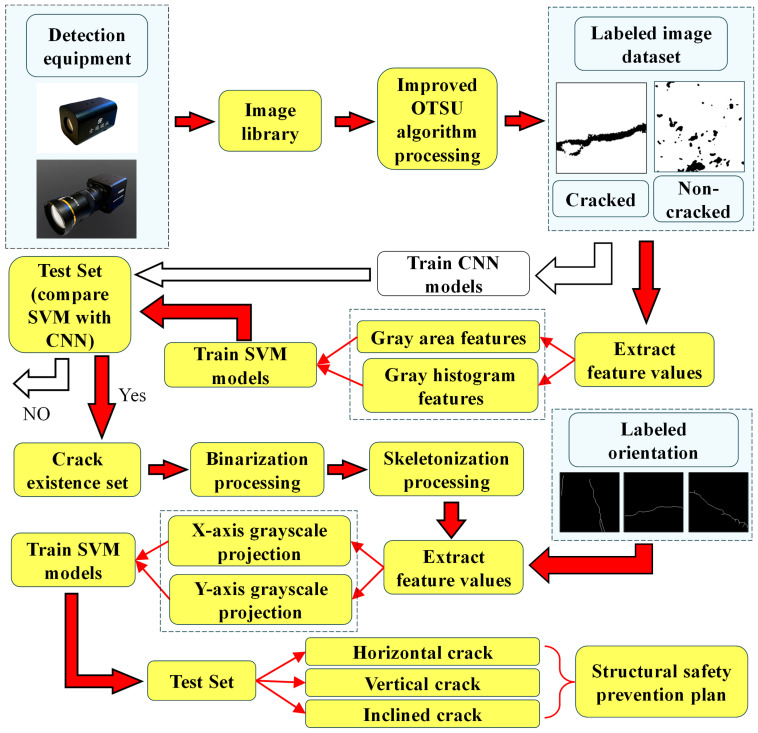
Workflow of Crack Detection and Classification Using Improved OTSU and SVM. Red arrows and yellow boxes represent the main workflow.

This study proposes a novel framework for crack recognition and classification, as illustrated in Figure 2 and Figure 3. As shown in Figure 2, the key image processing steps and training set construction process are presented, including the transformation from the original image to the binarized result, skeletonized representation, and the extraction of features for model training. The framework begins with the acquisition of images via detection equipment, which are aggregated into an image library. These images first undergo processing using an improved OTSU algorithm to generate a labeled dataset, distinguishing between cracked and non-cracked regions. For crack presence detection, two models are trained in parallel on the labeled dataset: a CNN model, which is trained directly on a labeled dataset consisting of two image groups—crack and non-crack—to classify images as cracked or non-cracked, and an SVM model trained on handcrafted features—including gray area and gray histogram information. A test set is then used to evaluate the performance of both models; if the validation is successful, the process proceeds, otherwise, model training is refined accordingly. Subsequently, images identified as containing cracks undergo binarization and skeletonization. Grayscale projections along the X-axis and Y-axis are performed on the skeletonized cracks to classify them into Horizontal, Vertical, or Inclined types. Finally, based on these classification results, a structural safety prevention plan is formulated, thereby completing a comprehensive loop from detection and identification to classification and application, which supports effective management of structural safety related to cracks.

## 4. Crack Detection and Classification

In this section, all experiments were conducted on a workstation equipped with an Intel Core i5-13400F CPU, 16 GB RAM, and an NVIDIA GeForce RTX 3070 Ti GPU. This unified hardware configuration ensures consistency, reproducibility, and fair comparison across all evaluated methods. The concrete crack images used in this study are sourced from the dataset available at https://www.kaggle.com/thesighsrikar/concrete-crack-images-for-classification (accessed on 9 April 2026).

### 4.1. Performance Comparison Between Classical and Improved OTSU Algorithms on Images with Different Resolutions

**Figure 4:**
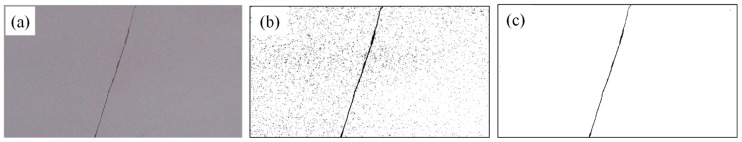
Classical OTSU and improved OTSU applied to high-resolution images: (**a**) original image, (**b**) result of classical OTSU, and (**c**) result of improved OTSU.

Figure 4 presents a comparison between the classical and improved OTSU algorithms applied to high-resolution images, including the original image, the result from the classical OTSU algorithm, and the result from the improved version. In high-resolution scenarios, the classical algorithm is prone to threshold deviation due to gray-level distortion caused by increased noise and complex background textures, resulting in significant noise in the processed image (Figure 4b). In contrast, the improved algorithm is more adaptable to complex scenes and effectively suppresses noise. Figure 4c shows that the improved OTSU algorithm significantly reduces noise and achieves higher segmentation accuracy.

**Figure 5:**
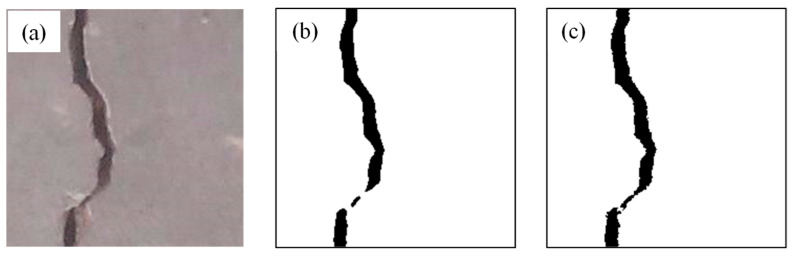
Classical OTSU and improved OTSU applied to low-resolution images: (**a**) original image, (**b**) result of classical OTSU, and (**c**) result of improved OTSU.

Figure 5 illustrates the performance of both algorithms on low-resolution images, including the original image, the result from the classical algorithm, and the result from the improved one. While the classical algorithm demonstrates reasonable noise suppression in low-resolution cases, it tends to lose fine crack features due to pre-denoising procedures. This issue arises because the algorithm typically applies a denoising step prior to segmentation, which enhances contrast between foreground and background but may weaken or even eliminate subtle crack structures. As a result, certain fine details are not preserved. The improved OTSU algorithm, by contrast, processes images directly without requiring pre-denoising, thereby achieving significant improvements in the preservation of crack details (Figure 5c). These results indicate that the improved OTSU algorithm, through optimized threshold selection, effectively addresses the noise interference problem in high-resolution images and overcomes the detail loss issue in low-resolution scenarios, thus demonstrating comprehensive advantages in high-precision crack extraction tasks.

### 4.2. Performance Analysis of Crack Detection Based on SVM and CNN

**Figure 6:**
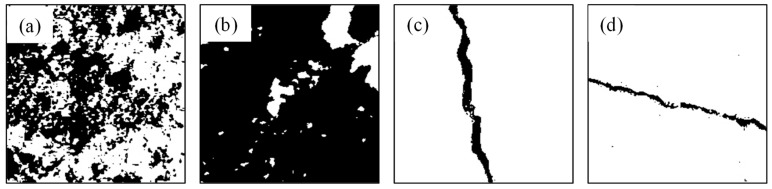
Unified processing results using improved OTSU Algorithm: (**a**,**b**) Processed images without cracks; (**c**,**d**) Processed images with cracks.

**Figure 7:**
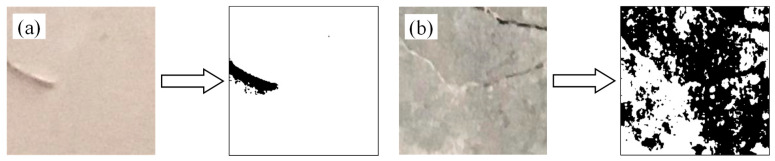
Examples of low grayscale contrast between cracks and background: (**a**) Improved OTSU processed image of a non-crack image; (**b**) improved OTSU processed image of a faint crack.

In the task of crack detection, the primary function of the SVM lies in classifying images based on extracted features, aiming to accurately distinguish crack regions from background or non-crack areas. Given the diverse morphological characteristics of cracks (e.g., linear, tortuous, branched), the effectiveness of feature extraction methods directly affects the classification performance of SVM. In this study, an improved OTSU algorithm is applied as a unified preprocessing step for all monitoring images to enhance contrast between crack regions and the background. This preprocessing enhances the clarity of crack features and improves the reliability of subsequent SVM training. As illustrated in Figure 6 (Unified processing results using improved OTSU algorithm), the visual distinction between crack and non-crack images becomes more pronounced after preprocessing. For feature selection, the SVM model employs crack area (a morphological feature) and grayscale histogram features as key discriminative indicators. A representative training set is constructed using both crack and non-crack images, and performance is validated on the constructed dataset. The experimental results indicate an overall classification accuracy exceeding 95%, demonstrating effective differentiation between crack and background in most scenarios. However, the model shows limitations when the grayscale contrast between cracks and background is low. Some low-contrast cracks are misclassified as non-crack regions, while certain background textures or noise are incorrectly identified as cracks. Typical misclassification cases are shown in Figure 7a,b, highlighting the adverse impact of background similarity on detection accuracy.

Following the SVM-based classification process, morphological operations are utilized to connect fragmented crack regions and extract complete contours, thereby providing a solid foundation for subsequent morphological analysis and structural assessment of cracks.

**Figure 8:**
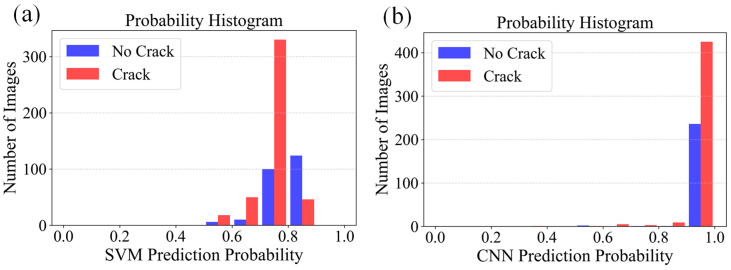
Prediction probability histograms based on improved OTSU-preprocessed images: (**a**) SVM and (**b**) CNN.

To comprehensively evaluate the performance of the algorithms, a comparative analysis was conducted between SVM and CNN for crack recognition under two preprocessing schemes, namely the improved OTSU algorithm and a combination of nonlinear denoising with the classical OTSU algorithm. All experiments were conducted on a unified dataset, which was randomly divided into training and testing sets with an approximate ratio of 80:20. The test set consists of 684 images, and the same data partition was used across all methods to ensure a fair comparison. Under the improved OTSU preprocessing, the SVM model achieved an accuracy of 97.92% for non-crack regions and 95.05% for crack regions, resulting in an overall accuracy of 96.05%. In comparison, the CNN model reached a non-crack recognition accuracy of 98.33% and a crack recognition accuracy of 94.02%, yielding an overall accuracy of 95.76%. The corresponding probability distributions are shown in Figure 8.

**Figure 9:**
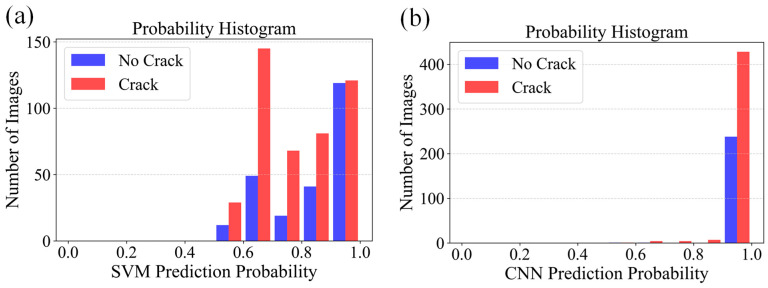
Prediction probability histograms based on classical OTSU-preprocessed images: (**a**) SVM and (**b**) CNN.

Under the preprocessing scheme combining nonlinear denoising with the classical OTSU algorithm, the performance of both models was further evaluated on the same dataset. The results are presented in Figure 9. For the SVM model, the non-crack recognition accuracy decreased to 92.50%, with a crack recognition accuracy of 86.71%, resulting in an overall accuracy of 88.74%. The CNN model, under the same conditions, achieved a non-crack recognition accuracy of 99.17%, a crack recognition accuracy of 94.37%, and an overall accuracy of 96.05%.

The results indicate that the improved OTSU preprocessing significantly enhances the performance of the SVM model, primarily due to its reliance on handcrafted features that are highly sensitive to feature quality. By improving the separation between crack and background regions, the proposed method produces more discriminative grayscale and morphological features, which directly benefits the SVM classifier. In contrast, the CNN model demonstrates relatively stable performance under different preprocessing schemes, as it is capable of automatically learning feature representations from raw image data and is therefore less sensitive to variations in preprocessing quality.

Furthermore, it is worth noting that, under the limited training data conditions considered in this study, the SVM model achieves performance comparable to that of the CNN model. This observation suggests that, when only a small number of labeled images are available-as is often the case in practical engineering scenarios-the combination of improved preprocessing and feature-based classification can provide a more efficient and robust solution. The enhanced binarization effect of the improved OTSU algorithm contributes to clearer structural representation of cracks, thereby reinforcing the effectiveness of SVM in small-sample conditions.

### 4.3. Optimization and Classification of Crack Detection Based on Skeletonization and Morphological Processing

Skeletonization, as a key morphological operation, aims to iteratively remove peripheral pixels of the target object, reducing the foreground region of an image to its central axis. This process effectively eliminates redundant details while preserving the geometric shape and topological connectivity of the object, thereby providing concise and essential morphological information for subsequent feature analysis, such as the extraction of crack length and branching structures. In the present study, skeleton extraction is primarily conducted using the Zhang-Suen algorithm.

The Zhang-Suen algorithm is a classical iterative method widely adopted in the field of skeletonization. Its core concept involves progressively removing non-essential boundary pixels through multiple iterations, ultimately preserving the skeletal structure that represents the medial axis of the object [35]. The algorithm operates on binary images, where a pixel set S is defined such that pixels with a value of 1 represent the foreground (e.g., crack regions), while those with a value of 0 correspond to the background. The algorithm follows a logical sequence in which edge pixels eligible for removal are identified and eliminated. In each iteration, isolated edge pixels—defined as pixels with all eight neighboring pixels equal to zero—are removed first. The remaining edge pixels are then evaluated for deletion based on specific criteria, such as the preservation of connectivity and the protection of terminal points. Pixels meeting all deletion conditions are removed, and this process continues until a stable skeleton structure is obtained. The iterative procedure relies on strict pixel deletion rules to ensure the continuity and topological consistency of the skeleton, making it well-suited for extracting the medial axes of elongated structures such as cracks.

(22) Delete(S(x,y))⇔(Condition 1)∧(Condition 2)

Here, Condition 1 and Condition 2 refer to specific configurations of neighboring pixels required to maintain structural integrity during the deletion process.

Based on the above morphological methods, this study establishes an optimized structural processing workflow for crack images, with the following steps: First, an improved OTSU algorithm is applied for binarization to extract the initial crack regions. Morphological bridging is then employed to enhance crack connectivity. This operation combines dilation and erosion (expressed as B = E(D(I,S),S), where I denotes the input binarized crack image, S represents the structuring element—typically a linear or elliptical kernel—D(I,S) and E(·) indicate the dilation and erosion operations, respectively, and B corresponds to the crack region after bridging). This approach enhances connectivity while minimizing edge blurring caused by dilation.

**Figure 10:**
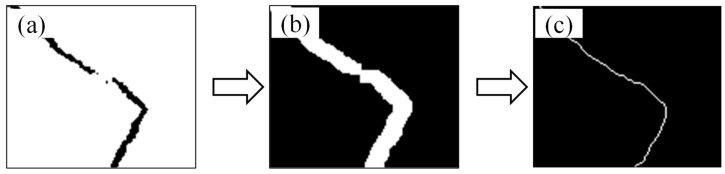
Morphological Processing for Crack Fracture Connection: (**a**) Original image processed with improved OTSU method; (**b**) Morphological connection of fracture openings; (**c**) Skeletonization through single-pixel refinement.

Subsequently, morphological hole-filling is applied to eliminate internal voids and improve crack integrity. This step is realized through morphological reconstruction (expressed as F = R(B, S), where R(B, S) denotes the reconstruction operation, and in practical computation, closing operation F = E(D(B, S),S), may be used). Skeletonization is then performed to extract the crack centerlines. Finally, morphological features and grayscale histogram features are integrated, and SVM is employed to classify crack types. The effectiveness of skeletonization and morphological bridging at fracture locations is illustrated in Figure 10.

**Figure 11:**
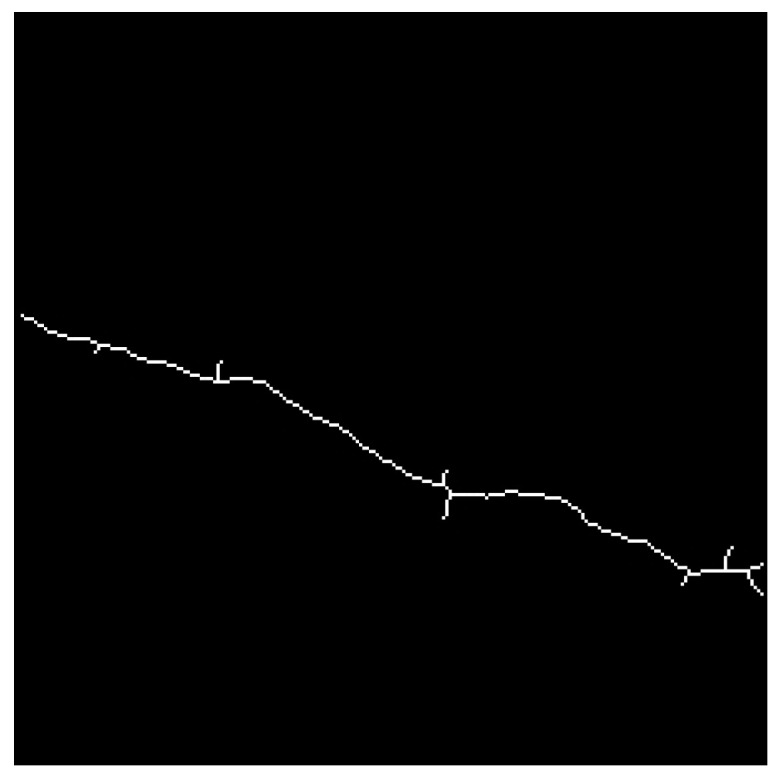
“Burr” effect after crack refinement.

In crack detection, dilation is used to fill voids, followed by erosion to restore the basic crack shape. The combined use of morphological bridging and filling is critical for improving crack extraction, as it enhances both connectivity and integrity, thereby contributing to more accurate feature extraction and classification. Appropriate selection of kernel size and shape enables the effective connection of discontinuities, background compression, and emphasis of crack features. However, dilation may also lead to thickened edges, burr-like artifacts, and grayscale distribution shifts [36], potentially affecting the accuracy of subsequent feature extraction and classification, as shown in Figure 11.

### 4.4. SVM-Based Classification of Crack Orientation

#### 4.4.1. Feature Extraction and Training Set Construction

Following the skeletonization process, grayscale projection techniques are applied to conduct in-depth analysis of crack morphology. By summing grayscale values along the X-axis or Y-axis of an image, the projection distribution of cracks in horizontal and vertical directions is obtained, revealing their geometric characteristics and spatial patterns. The X-axis projection represents the sum of pixel values in each column, while the Y-axis projection represents the sum in each row:

(23) $$P_{x}\left(j\right)\;=\;\sum_{j=1}^{H}I\left(i\cdot j\right),\;j\;=\;1,2,\cdot \cdot \cdot ,W$$

(24) $$P_{y}\left(i\right)=\sum_{i=1}^{W}I\left(i\cdot j\right),\;i=1,2,\cdot \cdot \cdot ,H$$

where $I\left(i\cdot j\right)$ denotes the grayscale value at the i-th row and j-th column of the image; H and W represent the image height and width, respectively; $P_{x}\left(j\right)$ and $P_{y}\left(i\right)$ are the grayscale projection values for the j-th column and i-th row, respectively.

**Figure 12:**
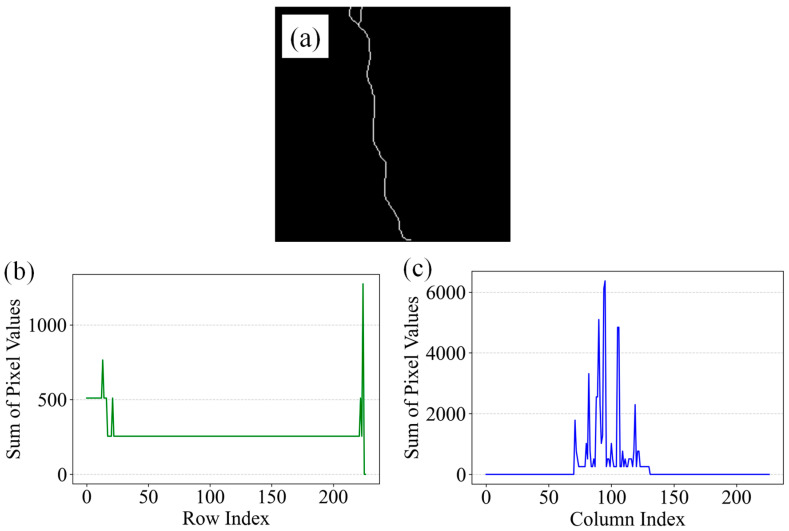
Grayscale distribution of a single-pixel crack: (**a**) crack image, (**b**) Y-axis projection, (**c**) X-axis projection.

Analysis of grayscale projections along the X and Y axes enables the automatic identification of crack orientation. For Vertically oriented cracks, most grayscale values in the X-axis projection approach zero, with significant peaks only at crack positions, while the Y-axis projection typically presents a single sharp peak. In contrast, horizontally oriented cracks produce the opposite pattern. Irregular or Y-shaped cracks result in multiphasic distributions on both axes, without clear zero-grayscale regions. Based on these characteristics, a grayscale trend-based classification criterion is proposed: vertical cracks are indicated by extensive zero-grayscale regions along the X-axis with a single peak on the Y-axis; horizontal cracks exhibit the inverse; Inclined cracks display no dominant peak in either axis. Taking a single-pixel-width crack as an example (see Figure 12), the X-axis projection shows near-zero values across most regions, with a sharp peak at the crack location, while the Y-axis projection contains a single prominent peak. This pattern aligns with the vertical crack criterion: extensive zero grayscale along the X-axis and a single peak along the Y-axis.

**Figure 13:**
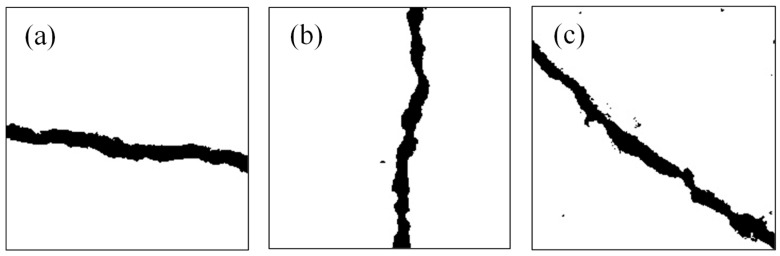
Crack types: (**a**) Horizontal crack, (**b**) Vertical crack, (**c**) Inclined crack.

To evaluate the performance of an SVM in classifying crack morphology and leverage its high accuracy in small-sample settings, a tailored classification approach is constructed:

- Feature extraction: Based on the skeletonized crack image, grayscale projection features along the X and Y axes are extracted. The X-axis projection reflects grayscale distribution in the horizontal direction, while the Y-axis projection corresponds to the vertical direction. The distinction between the two is used to differentiate Horizontal, Vertical, and Inclined cracks.
- Dataset construction: A total of 319 binarized crack images—derived from the improved OTSU algorithm—are used as the dataset, covering three representative crack types (as shown in Figure 13):

Figure 13a: Horizontal cracks extending along the horizontal axis, characterized by concentrated Y-axis projections and elongated X-axis distributions; 98 samples (30.72%);

Figure 13b: Vertical cracks developing along the vertical axis, characterized by concentrated X-axis projections and elongated Y-axis distributions; 109 samples (34.17%);

Figure 13c: Inclined cracks forming an angle with the coordinate axes, with both X and Y projections showing dispersed and continuous multiphasic features; 112 samples (35.11%).

- Model training: Based on the extracted features and labeled samples, an SVM classification model is constructed. By leveraging the generalization capability of SVM in small-sample scenarios, the model achieves the accurate classification of the three crack orientations, providing foundational support for subsequent quantitative crack analysis.

#### 4.4.2. SVM-Based Crack Classification Results

The SVM classifier employed in this study adopts a radial basis function (RBF) kernel with parameters C and γ. The input feature space is constructed based on the grayscale projection characteristics described in the previous section, from which statistical descriptors (i.e., mean and variance along both the horizontal (X) and vertical (Y) directions) are extracted to form a low-dimensional feature representation. This design enables the model to effectively capture orientation-related characteristics of cracks while maintaining robustness under limited data conditions.

Using these features, the SVM classifier was trained on the constructed dataset and subsequently evaluated on a validation set consisting of crack images processed by the improved OTSU method. This validation set covers a wide range of crack morphologies representative of practical structural conditions.

**Figure 14:**
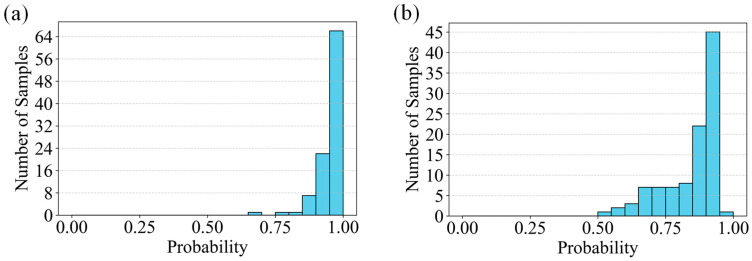

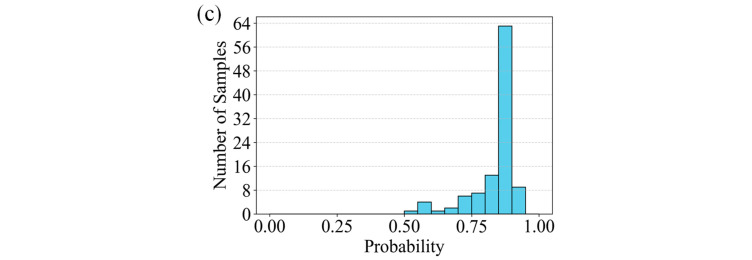
Confidence histograms of SVM classification results: (**a**) Horizontal cracks, (**b**) Vertical cracks, (**c**) Inclined cracks.

The updated classification results, summarized in Table 1 and visualized through the confidence histograms in Figure 14, indicate that the model exhibits strong discriminative capability across the three crack categories. The mean predicted probabilities achieved 97.25% for Vertical cracks, 96.08% for Inclined cracks, and 96.94% for Horizontal cracks, respectively. These values are reflected in the corresponding histogram distributions: for example, the Vertical crack category (Figure 14b) shows a pronounced aggregation of samples in the high-confidence region (approaching 1.0), demonstrating the stability and reliability of the predictions. A comparable pattern is observed for Horizontal cracks (Figure 14a), whereas Inclined cracks (Figure 14c) display a slightly broader confidence distribution, consistent with their marginally lower average probability.

The model achieves an overall classification accuracy exceeding 96%, with category-specific accuracies of 97.25%, 96.08%, and 96.94% for Vertical, Inclined, and Horizontal cracks, respectively. The predominance of high-confidence predictions across all categories confirms the robustness of the trained SVM model and validates its ability to effectively capture grayscale projection features and structural characteristics that differentiate crack orientations.

Further analysis of the misclassified and lower-confidence samples reveals that prediction uncertainty primarily arises from complex crack morphologies—such as branching, intersections, or irregular spatial distributions—and environmental interferences, including illumination variations and background texture noise. These factors are directly manifested in the histograms through the presence of samples distributed in the medium-to-low confidence ranges, indicating increased feature ambiguity under such conditions.

In summary, the combined tabular and graphical evidence demonstrates that the proposed SVM-based approach effectively distinguishes Vertical, Horizontal, and Inclined cracks with high accuracy and strong confidence consistency. The confidence histograms quantitatively capture the separability of the three categories within the feature space, while the updated metrics further substantiate the classifiers generalization capability. These findings highlight both the accuracy and interpretability of the method and provide a foundation for future enhancements aimed at improving robustness under complex crack geometries and challenging imaging environments.

#### 4.4.3. Performance Comparison Between SVM and CNN in Crack Classification

To further validate the effectiveness of the classification approach, CNN is employed for comparative analysis. A CNN model is designed and trained to perform automatic classification of crack orientation. Specifically, the CNN architecture consists of three convolutional layers followed by three max-pooling layers and one fully connected hidden layer. The convolutional layers use 3 × 3 kernels with ReLU activation functions, and the numbers of filters are set to 32, 64, and 128, respectively. Each convolutional layer is followed by a 2 × 2 max-pooling operation for spatial down sampling. After feature extraction, the feature maps are flattened and connected to a fully connected layer with 128 neurons, followed by a dropout layer with a rate of 0.5 to reduce overfitting. The output layer adopts a Softmax activation function with three neurons corresponding to Horizontal, Vertical, and Inclined crack categories. In terms of hyperparameters, the input images are uniformly resized to 227 × 227 pixels to ensure consistency with the SVM-based method, which is trained using the Adam optimizer with a learning rate of 0.001, a batch size of 32, and 20 training epochs.

**Figure 15:**
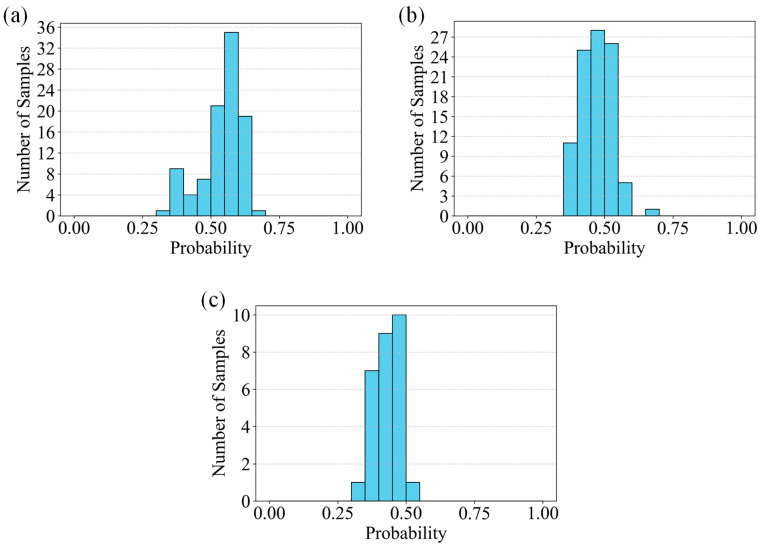
Confidence histograms of CNN classification results: (**a**) Horizontal cracks, (**b**) Vertical cracks, (**c**) Inclined cracks.

Table 2 presents the classification results of the CNN model, while Figure 15 provides corresponding confidence histograms, forming a multi-dimensional framework for evaluating model performance. The average confidence for Horizontal cracks reaches 94.90%, with the histogram in Figure 15a showing a concentration of samples in the high-probability interval (approaching 1.0), indicating strong classification confidence. For Vertical cracks, the average confidence is 87.16%, and Figure 15b reveals a distribution consistent with this value, though the presence of medium- and low-confidence samples contributes to a lower mean. For Inclined cracks, the average confidence is only 25.00%, as Figure 15c indicates a predominance of samples in the medium-to-low confidence intervals, visually reflecting the increased classification difficulty.

Compared with the SVM model, CNN shows slightly lower performance in Horizontal crack recognition, with 94.90% compared with 97.25%. The difference is more significant for Vertical cracks, where CNN achieves 87.16% compared with 96.94%, and especially for Inclined cracks, where CNN achieves 25.00% compared with 96.08%. These results indicate that CNN struggles to learn discriminative features under limited training data, particularly for cracks with subtle angular variations and ambiguous boundaries. This limitation is reflected in the larger proportion of medium- and low-confidence predictions. In contrast, the SVM model maintains robust performance by leveraging discriminative handcrafted features to construct an effective feature space. The dominance of high-confidence predictions demonstrates its strong generalization capability under small-sample conditions. Moreover, SVM provides better interpretability, as the extracted features, such as grayscale projection and morphological characteristics, have clear physical meanings and can be directly associated with crack geometry. In comparison, CNN features are learned automatically and are more abstract, making physical interpretation less straightforward. Both models demonstrate high computational efficiency, with inference time well below 0.1 s per image and negligible differences between them, indicating suitability for real-time tunnel inspection.

In summary, although CNN can achieve competitive performance in certain cases, its generalization is limited under small-sample conditions. SVM demonstrates greater stability and reliability in scenarios with limited data and clear feature representations, making it more suitable for practical crack classification tasks.

#### 4.4.4. Performance Comparison Between SVM and K-Means Clustering in Crack Classification

In the task of crack orientation classification, unsupervised learning methods such as clustering are widely applied in addition to supervised algorithms. Clustering identifies underlying data patterns by grouping samples based on feature similarity without requiring labeled information. K-Means, a commonly used clustering algorithm, partitions data by iteratively optimizing cluster centroids until convergence is achieved. This section compares the methodological differences between K-Means and SVM in the context of crack classification. The fundamental principle of K-Means clustering is defined as:

(25) $$\;J\;=\;\sum_{i=1}\sum_{x_{i}\in C_{i}}{\left\|x_{i}-u_{i}\right\|}^{2}$$

where k is the number of clusters, $C_{i}$ represents the i-th cluster, $u_{i}$ denotes the centroid of cluster $C_{i}$, and $x_{i}$ is a data point within cluster $C_{i}$ [37].

**Figure 16:**
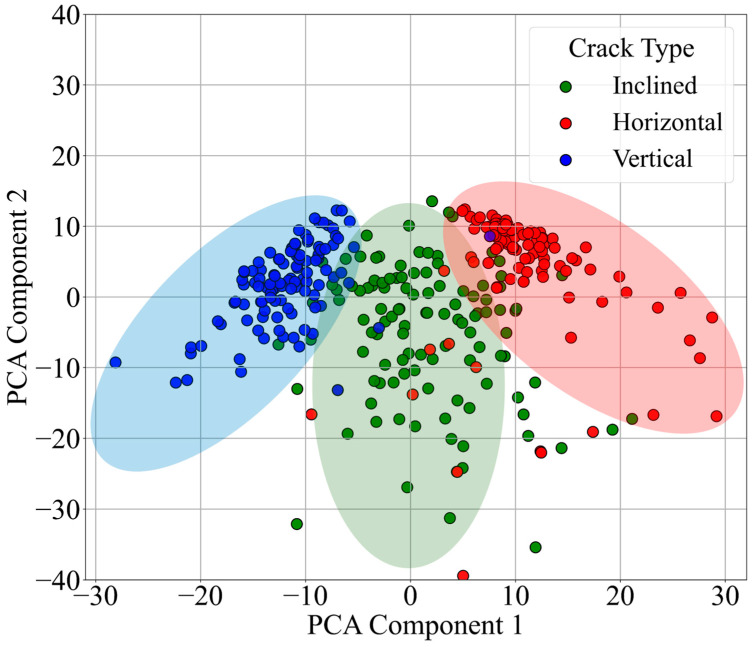
K-means dimensionality reduction clustering distribution map. Green, red and blue points/ellipses denote Inclined, Horizontal and Vertical cracks, respectively. Overlaps reflect spatial correlations in the feature space.

This study applies an unsupervised K-Means clustering approach to crack image recognition. A 456-dimensional feature vector is constructed based on grayscale image features. First, each image is resized to 227 × 227 pixels and converted to grayscale. Grayscale projection features are then extracted in both the Horizontal and vertical directions, along with the grayscale mean and variance. All feature vectors are subsequently standardized to eliminate the impact of differences in dimensional units. During training, the K-Means algorithm is applied to cluster three types of crack images: Horizontal, Inclined, and Vertical. The results of K-Means clustering after dimensionality reduction are shown in Figure 16. In the clustering outcome, Cluster 1 corresponds to the “Vertical Crack” category, Cluster 2 to the “Horizontal Crack” category, and Cluster 0 to the “Inclined Crack” category. In the prediction phase, the same features are extracted and standardized for the target image. The trained model is then used to predict the crack category, and the prediction confidence is estimated based on the inverse distance between the sample and the corresponding cluster center.

**Figure 17:**
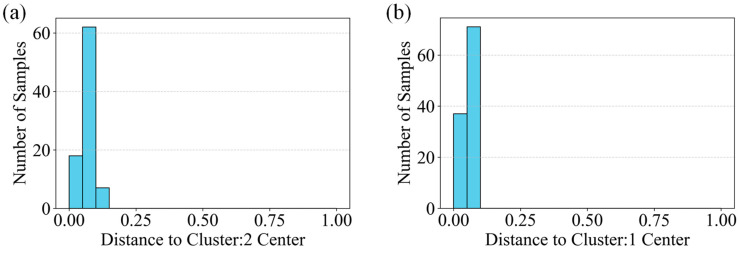

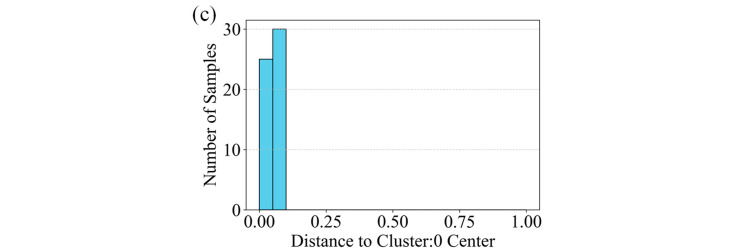
K-means Intra-cluster Distance Distribution Histograms: (**a**) Distance distribution from cluster center for Cluster 2 (Horizontal cracks), (**b**) Distance distribution from cluster center for Cluster 1 (Vertical cracks), (**c**) Distance distribution from cluster center for Cluster 0 (Inclined cracks).

The intra-cluster distance distribution histograms in Figure 17, along with the classification outcomes presented in Table 3, jointly validate the crack recognition characteristics of the K-Means clustering method. The degree of dispersion in the histograms reflects the varying clustering difficulties among different crack types. Specifically, samples of Horizontal cracks (Cluster 2) and Vertical cracks (Cluster 1) exhibit short distances to their respective centroids and relatively concentrated distributions (Figure 17a,b). These patterns correspond to the high average prediction probabilities of 98.17% and 88.78% in Table 3, indicating that grayscale projection features of regular-shaped cracks form clearly distinguishable clusters.

In contrast, Inclined cracks (Cluster 0) show larger and more scattered distances from the cluster center (Figure 17c), corresponding to a lower average prediction probability of 49.11%. This suggests that the inherent complexity of inclined crack orientations results in less cohesive grayscale feature distributions, complicating the clustering process.

In comparison to the SVM model, while K-Means can distinguish between distinctly shaped Horizontal and Vertical cracks, its reliance on unsupervised grayscale projection features limits its performance in classifying complex forms such as Inclined cracks. For instance, Inclined cracks with smaller angles may share similar grayscale features with Horizontal or Vertical cracks, leading to misclassification. The SVM model, by contrast, leverages supervised learning to target critical features, achieving an overall accuracy exceeding 96% (rising to 97% after excluding exceptional cases). Its average confidence level for Inclined cracks (96.08%) significantly outperforms that of K-Means (49.11%), demonstrating superior adaptability to crack shape diversity.

In summary, K-Means clustering is suitable for the preliminary differentiation of regular-shaped cracks. However, for more complex categories such as Inclined cracks, the SVM model—based on supervised learning and focused feature extraction—offers irreplaceable advantages in both precision and robustness. These findings provide empirical support and methodological guidance for model selection and optimization in intelligent crack recognition tasks.

#### 4.4.5. Performance Comparison Between SVM and ResNet in Crack Classification

To further evaluate the performance of deep learning methods in crack classification tasks, the ResNet-18 model was introduced for comparative analysis. The ResNet-18 model pre-trained on ImageNet was adopted as the deep learning baseline. To ensure a fair comparison with the SVM-based method, all input images were uniformly resized to 227 × 227 pixels. The model was fine-tuned using a cross-entropy loss function and optimized with the Adam optimizer, with an initial learning rate of 1 × 10^−4^, a batch size of 16, and a total of 20 training epochs. All experiments were implemented in PyCharm (version 2024.2.4) and conducted on a GPU to ensure computational efficiency. The final fully connected layer was modified to output three classes corresponding to Horizontal, Vertical, and Inclined cracks.

**Figure 18:**
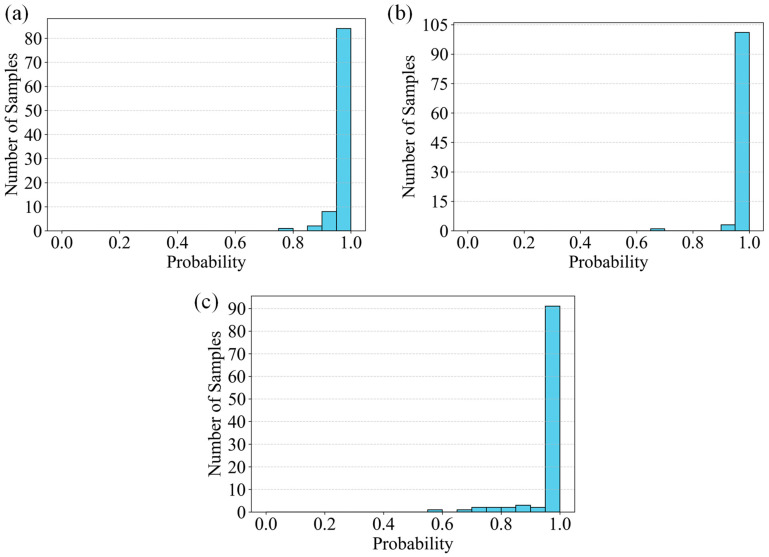
Confidence histograms of ResNet classification results: (**a**) Horizontal cracks, (**b**) Vertical cracks, (**c**) Inclined cracks.

Table 4 presents the classification results of the ResNet-18 model, while Figure 18 provides the corresponding prediction probability histograms, forming a multi-dimensional framework for evaluating model performance. In terms of average prediction probability, the model achieves 96.94% for Horizontal cracks, with Figure 18a showing a clear concentration of samples in the high-probability interval (close to 1.0), reflecting high classification stability. For Vertical cracks, the average prediction probability is 96.33%, and Figure 18b reveals a distribution aligned with this value, demonstrating consistent confidence across most samples. For Inclined cracks, the average prediction probability is 92.88%, and Figure 18c indicates a slight shift toward medium-probability intervals compared to the other two categories, visually confirming the greater challenge of feature extraction for this type of crack. The overall classification accuracy of the model reaches 95.38%, further verifying its reliable performance in crack orientation classification.

Compared with the SVM model, ResNet-18 shows slightly lower performance in recognizing Horizontal cracks (96.94% vs. 97.25%) and Vertical cracks (96.33% vs. 96.94%), although the differences are relatively small. This indicates that deep learning models can achieve strong discriminative capability for categories with clear structural features. However, for Inclined cracks, ResNet-18 (92.88% vs. 96.08%) still underperforms compared to SVM, suggesting reduced stability when dealing with samples exhibiting subtle angular variations and indistinct boundaries.

The prediction confidence of the ResNet-18 model is generally above 90%, indicating that the model has learned relatively stable feature representations. However, despite the high confidence, its classification accuracy—especially for Inclined cracks—remains lower than that of SVM. This suggests that deep learning models may struggle to effectively generalize fine-grained orientation features under limited data conditions. In contrast, the SVM model, based on discriminative handcrafted features, is less sensitive to data scale and can better capture subtle differences, resulting in more stable performance in small-sample scenarios.

In summary, ResNet-18 exhibits strong overall performance in crack classification tasks and achieves results comparable to SVM in most categories. However, under small-sample and complex-feature scenarios, its performance remains slightly inferior to that of SVM. These findings further indicate that, when the dataset size is limited, traditional machine learning methods based on discriminative feature engineering, such as SVM, still hold significant advantages, whereas deep learning approaches demonstrate greater potential when sufficient training data are available.

## 5. Example Validation

**Figure 19:**
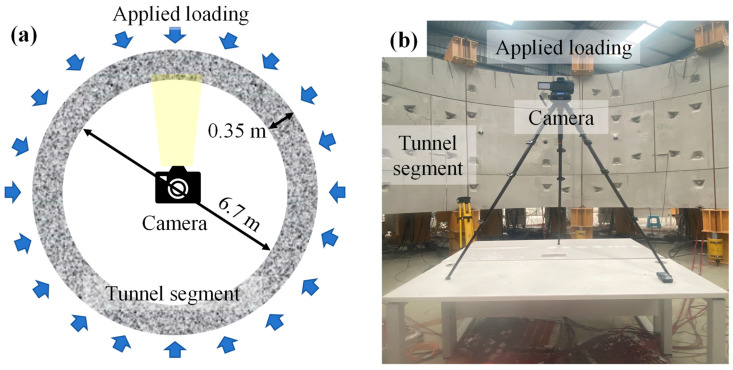
Schematic diagram and physical test setup of (**a**) tunnel segment loading and (**b**) crack image acquisition system.

Crack orientation emerges as a key morphological feature that directly encodes the underlying mechanical response of the lining structure. Unlike crack width or length, which only reflect the severity of damage, orientation reveals the specific stress states that initiate and drive crack development in segments. In this context, full-scale laboratory segment loading tests were conducted, and synchronous imaging of cracks was performed to facilitate subsequent quantitative analysis. This experimental investigation is centered on the conventional shield tunnel employed in Metro tunnel, characterized by an outer diameter of 6.7 m, with its constituent segments featuring a thickness of 0.35 m. Throughout the experiment, external loading was imparted to the segment assembly (illustrated in the schematic layout of Figure 19a and the physical test setup of Figure 19b), while a camera system was deployed to acquire surface crack images of the segments. A total of 22 crack image samples were collected, which were subsequently utilized to evaluate the practical performance of SVM-based crack orientation classification framework.

Table 5 presents the crack classification results under real-world conditions, covering three typical crack categories: Vertical, Inclined, and Horizontal. Specifically, vertical cracks account for the largest proportion of the test samples (54.54%, *n* = 12), with an average prediction probability of 91.67%. By contrast, inclined cracks (*n* = 5, 22.73%) and horizontal cracks (*n* = 5, 22.73%) both achieve a 100% average prediction probability, indicating high model confidence in identifying these two crack types. Overall, the proposed method yields an excellent classification accuracy of 95.45% in practical scenarios, which verifies its strong robustness and applicability for on-site crack detection tasks.

**Figure 20:**
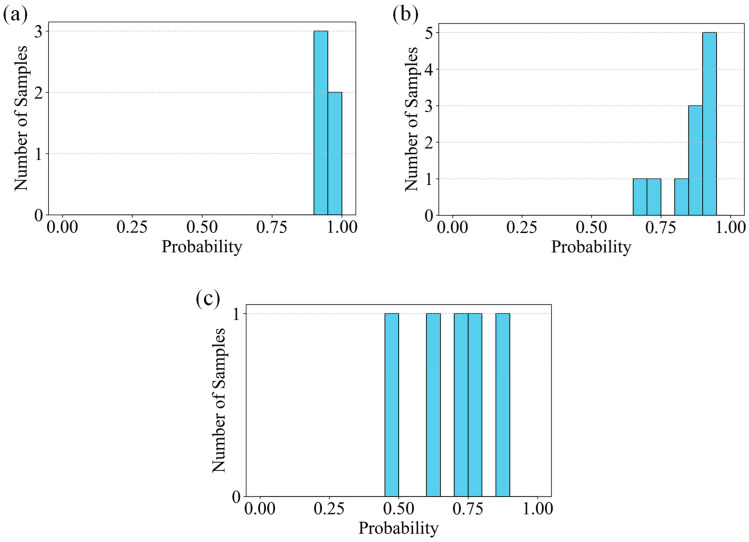
Confidence Histograms of SVM Classification Results Under Real-World Conditions: (**a**) Horizontal Cracks, (**b**) Vertical Cracks, (**c**) Inclined Cracks.

To intuitively evaluate the classification performance of the SVM model under real-world conditions, Figure 20 plots the confidence histograms of the SVM classification results, which are further divided into three subgraphs based on crack types: (a) Horizontal cracks, (b) Vertical cracks, and (c) Inclined cracks. These histograms clearly reflect the distribution characteristics of the classification confidence scores output by the model across different crack categories, providing a quantitative basis for subsequent model accuracy assessment and optimization.

**Figure 21:**
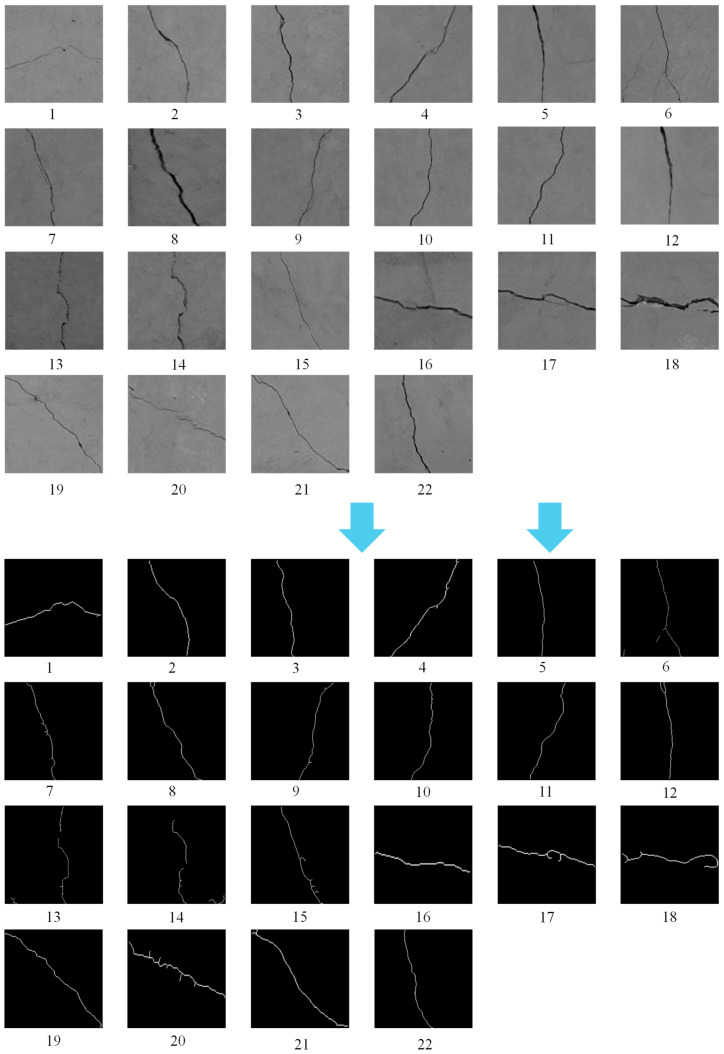
Collection of 22 in situ crack images acquired during field testing for orientation recognition. Numbers denote sample indices; arrows mark representative samples. Upper row: original images; lower row: segmented binary images.

The images obtained after applying skeletonization and other morphological processing techniques to the field crack photographs are shown in Figure 21. These operations simplify the crack contours into single-pixel-width skeletons while preserving the geometric features (e.g., direction, branching) of the original cracks, providing a streamlined representation for subsequent orientation classification tasks. The dataset covers diverse crack patterns (vertical, inclined, and irregular forms), which align with the real-world crack distribution characteristics of tunnel segments in the test.

**Figure 22:**
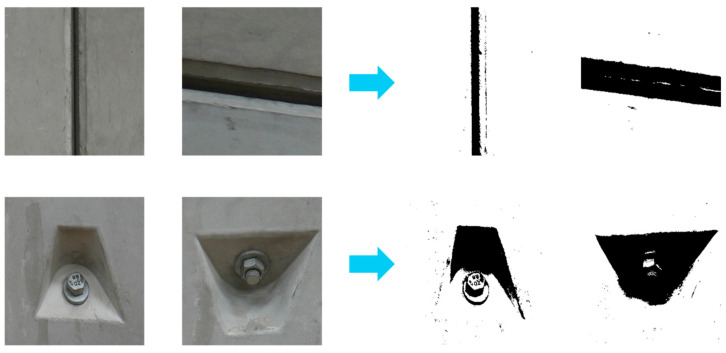
Binarization results of representative crack-like objects after improved OTSU processing.

To further evaluate the robustness of the proposed method in complex scenarios, additional experiments were conducted on images containing crack-like objects, such as joints, construction marks, bolt holes, drill holes, and segment seams. These objects are commonly found in tunnel linings and may introduce interference in crack detection tasks. As shown in Figure 22, the improved OTSU algorithm is first applied to these images to obtain binarized results. Although some structures exhibit visual similarity to cracks, their morphological characteristics differ from actual crack patterns. Subsequently, the processed images are fed into the SVM model for classification.

**Figure 23:**
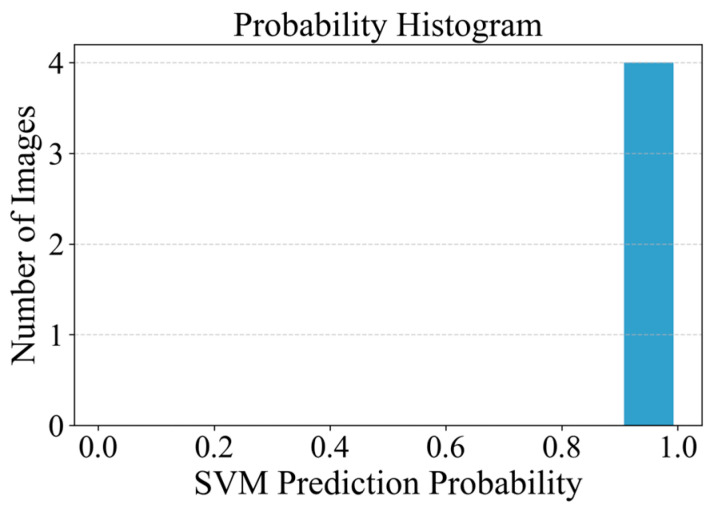
SVM prediction confidence for crack-like object images classified as non-crack.

The corresponding prediction results are presented in Figure 23. It can be observed that the SVM model correctly classifies these crack-like objects as non-crack images with high confidence values (close to 1.0). This demonstrates that the proposed framework is capable of filtering out certain types of interference and reducing false positives in crack detection. However, it should be noted that the number of such samples in this study is relatively limited. Further investigation with a more diverse dataset is necessary to comprehensively evaluate the generalization ability of the model in more complex real-world environments.

## 6. Conclusions

This study systematically explores crack detection and classification methods based on machine vision and deep learning. Through in-depth research on image preprocessing algorithms, classification models, and engineering applications, the following key conclusions are drawn:

(1) By integrating global threshold calculation with local grayscale variance analysis and dynamic threshold adjustment, the algorithm achieves superior performance in both high and low-resolution crack images. It not only suppresses noise interference in high-resolution images with complex backgrounds but also avoids the loss of fine crack details in low-resolution images caused by pre-denoising, laying a reliable foundation for subsequent crack feature extraction and classification. In this study, a 5 × 5 sliding window is adopted for 227 × 227 pixel images, which achieves a good balance between segmentation performance and computational efficiency.
(2) In the comparative analysis of crack classification models, the SVM model demonstrates clear advantages under small-sample conditions, achieving an overall accuracy above 96% based on grayscale projection and morphological features. In contrast, deep learning models show performance degradation with limited training data. The CNN model performs poorly for Inclined cracks, with confidence dropping to 25.00%, while ResNet-18 achieves relatively stable performance with an overall accuracy of 95.38% but remains less effective than SVM for complex crack orientations. The K-means algorithm exhibits the lowest accuracy of 78.05% due to its inability to capture discriminative features without supervision. Overall, SVM proves to be more suitable for engineering scenarios with limited labeled data, whereas deep learning methods show greater potential when sufficient data are available.
(3) Verification in shield tunnel segment loading tests shows that the framework achieves an overall classification accuracy of 95.45% in real-world conditions, with 100% average prediction confidence for Horizontal and Inclined cracks. This verifies the framework’s strong robustness and practical applicability, providing effective technical support for analyzing the mechanical response of tunnel segments through crack orientation features. In addition, validation on crack-like objects (e.g., joints, bolt holes, and segment seams) shows that the proposed method can effectively distinguish them from actual cracks, demonstrating its ability to reduce false positives in complex tunnel environments.

Despite the promising results, several limitations remain. The relatively small dataset may restrict generalization under more complex real-world conditions, and the classification performance still depends on handcrafted features, limiting adaptability to irregular patterns. In addition, the current framework focuses on crack orientation classification and does not include pixel-level segmentation or multi-scale analysis. Future work will focus on expanding the dataset, improving robustness in complex environments, and integrating deep learning or hybrid approaches to enhance feature representation and generalization. Extending the method to more comprehensive crack characterization, such as width estimation and severity assessment, will also be explored.

## Figures and Tables

**Table 1:** Crack Classification Results Using the SVM Algorithm.

Category	Quantity	Proportion	Average Predicted Probability	Overall Accuracy
Vertical crack	109	34.17%	97.25%	96.76%
Inclined crack	112	35.11%	96.08%	
Horizontal crack	98	30.72%	96.94%	

**Table 2:** Crack classification results using the CNN algorithm.

Category	Quantity	Proportion	Average Predicted Probability	Overall Accuracy
Vertical Crack	109	34.17%	87.16%	67.71%
Inclined Crack	112	35.11%	25.00%	
Horizontal Crack	98	30.72%	94.90%	

**Table 3:** K-Means Clustering Results for Crack Classification.

Category	Quantity	Proportion	Average Prediction Probability	Overall Accuracy
Vertical Crack	109	34.17%	98.17%	78.05%
Inclined Crack	112	35.11%	49.11%	
Horizontal Crack	98	30.72%	88.78%	

**Table 4:** ResNet Results for Crack Classification.

Category	Quantity	Proportion	Average Prediction Probability	Overall Accuracy
Vertical Crack	109	34.17%	96.33%	95.38%
Inclined Crack	112	35.11%	92.88%	
Horizontal Crack	98	30.72%	96.94%	

**Table 5:** Real-world conditions Results for Crack Classification.

Category	Quantity	Proportion	Average Prediction Probability	Overall Accuracy
Vertical Crack	12	54.54%	91.67%	95.45%
Inclined Crack	5	22.73%	100%	
Horizontal Crack	5	22.73%	100%	

## Data Availability

The source code and figures presented in this paper are available from the corresponding author upon reasonable request.
